# Keep moving and stay in a good shape to find your homologous recombination partner

**DOI:** 10.1007/s00294-018-0873-1

**Published:** 2018-08-10

**Authors:** Hélène Bordelet, Karine Dubrana

**Affiliations:** Laboratoire Instabilité et Organisation Nucléaire, iRCM, IBFJ, DRF, CEA. 2 INSERM, U967. 3 Université Paris Diderot et Paris Saclay, UMR967, Fontenay-aux-roses, 92265 France

**Keywords:** Homologous recombination, Homology search, DSB, Resection, Heterochromatin, Nuclear organization

## Abstract

Genomic DNA is constantly exposed to damage. Among the lesion in DNA, double-strand breaks (DSB), because they disrupt the two strands of the DNA double helix, are the more dangerous. DSB are repaired through two evolutionary conserved mechanisms: Non-Homologous End Joining (NHEJ) and Homologous Recombination (HR). Whereas NHEJ simply reseals the double helix with no or minimal processing, HR necessitates the formation of a 3′ssDNA through the processing of DSB ends by the resection machinery and relies on the recognition and pairing of this 3′ssDNA tails with an intact homologous sequence. Despite years of active research on HR, the manner by which the two homologous sequences find each other in the crowded nucleus, and how this modulates HR efficiency, only recently emerges. Here, we review recent advances in our understanding of the factors limiting the search of a homologous sequence during HR.

## Introduction

Genome stability implies that DNA damage, arising from either environmental stress or from endogenous events is robustly dealt with. Among DNA lesions, DNA double-strand breaks (DSB) are particularly deleterious. A single DSB can be lethal if unrepaired, particularly in a haploid organism such as yeast, and may lead to loss of genetic information and chromosome rearrangements if repaired improperly. Consequently, failure to repair DNA damages accurately leads to cancer and other diseases of ageing. From yeast to human, two conserved pathways for DSB repair are active: Non-Homologous End Joining (NHEJ) that simply religates DSB extremities, and Homologous Recombination (HR) that needs to copy an intact homologous sequence to reconstitute the broken site.

DNA DSBs are initially sensed and independently bound by the KU heterodimer and the MRX complex (Mre11–Rad50–Xrs2; Mre11-Rad50-Nbs1, MRN in humans). This initial binding helps recruit the NHEJ ligase, Lig4 which ligates DSB extremities even in the absence of significant homology. If not ligated by NHEJ, DSB is processed to generate 3′ single strand overhangs by partially redundant nucleases, which include Mre11/Sae2, Dna2 and Exo1 (Mimitou and Symington 2008; Cejka 2015; Symington 2016). The resulting 3′ss overhangs generated by the concerted action of MRX/Sae2, Exo1 and Sgs1/Dna2 proteins are rapidly stabilized by RPA. RPA, in turn, recruits proteins of the Rad52 epistasis group, such as Rad51, and these carry out strand invasion of the homologous template (Shinohara et al. 1992; Sung 1994; Baumann et al. 1996; Fortin and Symington 2002). New DNA synthesis copying the invaded duplex seals the DSB and after the resolution of the recombination intermediate structures, two intact DNA duplexes are restored (Symington et al. 2014; Wright et al. 2018).

HR can be separated into various sub-pathways that have different consequences for genome stability. HR is often considered to be an error-free mechanism for the repair of DSBs, as two-ended breaks are repaired primarily by Gene Conversion (GC) using a homologous duplex as template. However, if the donor sequence is not entirely homologous, GC can lead to loss of heterozygosity (LOH). Following HR repair, LOH is usually restrained to a small region surrounding the DSB but can be more extensive if HR occurs by Break Induced Replication (BIR). Indeed, during BIR repair the initial Displacement loop (D-loop) strand invasion event is followed by the establishment of a processive replication fork. This DNA synthesis can continue for hundreds of kb to the end of the chromosome, resulting in a long track of LOH (Llorente et al. 2008; Kramara et al. 2018). BIR is the repair of choice when only one DSB end is available for strand invasion, but is also thought to be at play to restart collapsed replication forks and to elongate telomeres that are lost in the absence of telomerase or when telomeres are uncapped (Lundblad and Blackburn 1993; McEachern and Haber 2006; Llorente et al. 2008).

In all cases, a prerequisite to HR repair is the encounter of the broken and homologous sequences. Although the sister chromatid, which shares perfect homology and is held in close proximity by the cohesin complex is the evident template for recombination, homologous sequences present on either the homologous chromosome (allelic recombination) or on a non-homologous chromosome (ectopic recombination) can also be used. Then, two scenarios can be envisioned, one in which the homologous sequences are in proximity before DNA damage and a second in which the homologous sequence is actively searched by the broken molecule. Recent results indicate that the truth lies in between with a post damage pairing resulting from both diffusion and chance of encounters limited by pre-existing distance. As a consequence, the homology search process is largely impacted by chromatin mobility and chromosomes position in the nucleus. However, recent evidence indicates that mobility and distance between homologous loci are not the sole limiting factors for HR.

Here, we review recent advances in our understanding of the factors limiting the search of a homologous sequence during HR. We focus on the molecular steps that have been shown to limit the homology search process and present recent advances in our understanding of how the chromosomal context of the DSB, its nuclear localization and its chromatin status impact on the success of recombination processes.

### Finding a homologous sequence is a challenge in the nuclear context

Over the last years, both microscopy and chromosome conformation capture experiments have revealed the non-random positioning of chromosomes in the nucleus. Although the patterns of this organization differ among eukaryotes, they impose constraints on the distances between sequences in the nucleus.

In haploid budding yeast, the tethering of the 16 centromeres at one pole and the gathering the 32 telomeres among 3–4 foci at the nuclear periphery imposes a Rabl-like conformation during exponential growth (Taddei et al. 2010; Albert et al. 2012). This organization largely favours interaction between centromeric proximal sequences as well as contact between subtelomeres (Bystricky et al. 2005; Schober et al. 2008; Therizols et al. 2010; Duan et al. 2010; Agmon et al. 2013). Consistently, recombination between centromeres on one hand and between subtelomeric sequences on the other hand occurs efficiently (Burgess and Kleckner 1999; Brown et al. 2010; Agmon et al. 2013; Lee et al. 2016).

In mammals, chromosomes occupy distinct chromosome territories that intermingle only little with each other in accordance with scarce interchromosomal interactions which may disfavour ectopic recombination (Cremer and Cremer 2010; Rao et al. 2014). In most diploid organisms, with the exception of Dipterans, homologous chromosomes are apart in somatic cells (Cremer et al. 2001; Lorenz et al. 2003; Rong and Golic 2003; Joyce et al. 2016). Noteworthy, in human cells, homologous chromosomes are even more distant than what could be expected based on the known rules of chromosomes radial organization in the nucleus and probably less likely to recombine (Heride et al. 2010).

Thus, for both ectopic recombination and HR between homologs, finding the right donor sequence in the 3D space of the nucleus is the first challenging task in HR. Homology search has indeed early on been shown to be a rate-limiting step for recombination in budding yeast and proposed to occur through random 3D collisions rather than sliding along the DNA (Wilson et al. 1994). Although this study clearly gave us hints about the mechanism of homology search, because it used plasmids or linearized DNA for monitoring recombination, it might not reflect physiological recombination events. Indeed, these free DNA molecules have been shown to differ in their mobility from chromosomic sites (Gartenberg et al. 2004).

### Increasing mobility: a functional requirement for homology search?

Chromosomic DNA motion is constrained in all organisms, not only by the polymeric nature of the chromatin fibre and by its confinement in the nucleus but also by the interaction of the chromosomes together and with nuclear structures, such as the nuclear membrane (Chubb et al. 2002; Zimmer and Fabre 2011; Hajjoul et al. 2013; Vasquez and Bloom 2014; Bronshtein et al. 2015; Marshall and Fung 2016).

In *S. cerevisiae*, the chromosomic DNA motion is limited by the tethering of centromeres and telomeres to nuclear membrane components (Hediger et al. 2002; Winey and Bloom 2012; Verdaasdonk et al. 2013; Strecker et al. 2016). This constraint is at least partially relieved in response to DNA damage. Indeed, the broken site mobility was shown to increase four- to fivefold and mobility of other sites in the genome also elevates, albeit to a lesser extent (Dion et al. 2012; Miné-Hattab and Rothstein 2012). Both global and local increase in motion depend on checkpoint activation and diverse, non-exclusive, mechanisms have been proposed (for a Review Smith and Rothstein 2017; Zimmer and Fabre 2018). First, a checkpoint-mediated disruption of centromeres anchoring has recently been proposed to participate in this *DSB-*induced chromatin mobility (Strecker et al. 2016). Second, a change in chromatin stiffness caused by both local and global chromatin remodelling may account for increased motion (Hauer et al. 2017). The increase of DSB ends mobility also depends on the recombination proteins Rad51, Rad54, and has thereby been proposed to promote recombination by allowing efficient scanning of the genome to find the appropriate template for repair (Dion et al. 2012; Miné-Hattab and Rothstein 2012). In agreement with this hypothesis, the targeting of the Ino80 chromatin remodelling factor that enhances locally chromatin mobility concomitantly increases spontaneous recombination between non-allelic homologous sequences (Neumann et al. 2012). However, this correlation could stem from Ino80-mediated effects other than mobility increase. The fact that some mutants affected in DSB-induced mobility show no defect in HR efficiency further questions this functional relationship (Lee et al. 2016; Strecker et al. 2016).

Increased DSB mobility has also been observed in mammals but remains controversial (Dion and Gasser 2013; Lemaître and Soutoglou 2015). On the one hand, several findings are similar to yeast where damaged chromatin displays a twofold increased mobility compared to unbroken DNA (Dimitrova et al. 2008; Krawczyk et al. 2012; Lottersberger et al. 2015). In this case, enhanced mobility requires 53BP1 and the INM proteins SUN1 and SUN2 (Dimitrova et al. 2008; Lottersberger et al. 2015). On the other hand, other studies have observed that chromatin containing DSBs exhibits limited mobility (Kruhlak et al. 2006; Soutoglou et al. 2007; Jakob et al. 2009). This apparent discrepancy may be attributed to the type of damage incurred and/or to the way it activates the ATM/ATR checkpoint, which was shown in yeast to contribute to DSB motion (Seeber et al. 2013). Consistently, ATM mediates DSB mobility and relocation of proximal DSBs to a repair centre in mammalian cells (Neumaier et al. 2012; Becker et al. 2014; Caron et al. 2015). A recent study in human cells revealed that the actin-nucleating Arp2-3 complex that promotes nuclear actin filament polymerisation is one of the driver of the motion of DSBs engaged in HR (Schrank et al. 2018). This sustains that in human cells as well, an active mechanism that promotes DSB movement participates in homology search. The same Arp2-3 complex is also the driver of heterochromatic DSB extrusion and perinuclear relocalisation in Drosophila (Caridi et al. 2018). Finally, the actin cytoskeleton seems to contribute to chromatin mobility in yeast but its requirement for DSB mobility remains to be tested (Spichal et al. 2016). Overall, the conservation of this mechanism in various species plead in favour of its functional relevance for DNA repair.

Although the modulation of chromosome mobility may enhance homology search efficiency, it is clearly not sufficient to overcome the constraints imposed by chromosome organization. Indeed, several studies in yeast recently showed that recombination efficiency decreases with the spatial distance between a DSB and its homologous targets (Burgess and Kleckner 1999; Agmon et al. 2013; Lee et al. 2016; Batté et al. 2017). Accordingly, sequences actively brought into close proximity either using the MAT Recombination Enhancer (Li et al. 2012; Mehta et al. 2017) or clustering subtelomeric sequences (Batté et al. 2017) recombine more efficiently. Thus, the physical distance between the DSB and the recombination donor is a limiting factor for HR success. The fact that recombination efficiency and spatial distance anti-correlate also implies that there is a time limit for homology search.

### Resection, a ticking clock for homology search?

The time limiting homology search may be imposed by the rate of resection. Despite the fact that some resection is required to unmask ssDNA that will search for a homologous sequence, extensive resection has also been shown to ultimately lead up to the loss of the DSB proximal sequences required for recombination.

DSB resection occurs through the concerted action of conserved redundant nucleases including the MRX^MRN^ complex, Exo1 and the Dna2/Sgs1^BLM/WRN^ complex. The MRX^MRN^–Sae2^CtIP^ complex first catalyses an endonucleolytic cleavage in the 5′ strand that frees a 3′ extremity used as an entry point for the degradation of the DNA toward the DSB end by the 3′–5′ exonuclease activity of Mre11 (Cannavo and Cejka 2014). More extensive resection in the 5′–3′ direction is taken over by the processive complexes Exo1 and Sgs1–Dna2 (for a review Symington and Gautier 2011). The 3′ssDNA formed is first coated by RPA and then by the Rad51 recombinase forming a filament that will engage the search for a homologous sequence. This search, if successful, will be followed by the strand invasion of the template sequence, DNA polymerization and resolution of the D-loop structure to restore intact duplex DNA (Symington et al. 2014; Wright et al. 2018).

At the biochemical level, the length of the homologous sequence coated by bacterial Rad51 ortholog RecA is a crucial factor in the rate-limiting step of homologous pairing in vitro (Forget and Kowalczykowski 2012). This possibly reflects a stabilisation of the duplex by the number of paired bases but could also result from the fact that the recombination filament is able to simultaneously bind multiple non-contiguous sequences. This has been observed in vitro for the RecA filament (Forget and Kowalczykowski 2012) and more recently in vivo in *S. cerevisiae* (Piazza et al. 2017). Although these observations explain that a certain amount of resected DNA is required for efficient homology search in vivo, the fact that slowing down resection can increase recombination efficiency at subtelomeric and some intrachromosomic DSBs (Lee et al. 2016; Batté et al. 2017) suggests that too much resection can also limit HR.

A possible rationale for these observations may be brought by the unstable nature of the 3′ssDNA overhang. Indeed, a study by the Diffley’s laboratory showed that once formed by resection, the ssDNA is stable only few hours and later lost (Zierhut and Diffley 2008). Based on recent data, one can envision at least two mechanisms to account for the processing of the 3′ssDNA  overhang. One is linked to the exhaustion of the RPA protein that can be caused by hyper-resection or uncontrolled replication and will expose unprotected ssDNA to the formation of secondary structures and enzymatic processing leading to its degradation (Chen et al. 2013; Toledo et al. 2013). Consistently, overexpressing subunits of the RPA complex increases the recombination efficiency (Lee et al. 2016). The second mechanism, which will depend on the local sequence context, is linked to the propensity of the ssDNA if bearing repeated elements to invade multiple sequences. These multi-invasions would form substrates for nucleases such as the 3′-flap Rad1-Rad10 nuclease leading to the processing and attrition of the 3′ssDNA overhang (for a review Lyndaker and Alani 2009). These competing strand invasions are particularly likely to occur at subtelomeres that harbour multiple repeated elements (X core, Y’...) and could account for the rapid loss of telomere proximal sequences previously observed at subtelomeric DSBs (Batté et al. 2017). Parasitic recombination events probably also occur at intrachromosomic DSB flanked by multi-genes family or Ty elements (Jain et al. 2016). Therefore, the chromosomal environment and especially repeated elements can render DSB flanking sequences more or less prone to resection. This, together with the fact that the genetic information on the 3′ overhang is lost concomitantly with 5′ resection, explains how resection limits homology search and HR repair. It also provides a rationale to explain how increasing the size of the homology improves HR (Lee et al. 2016).

Excessive resection does not only limit homology search, but also affects repair outcomes. Indeed, in the context of a subtelomeric DSB, the telomeric proximal side of the break is very sensitive to resection and is rapidly lost preventing gene conversion, a mechanism that requires both DNA ends. The DSB can then only be repaired by break-induced replication (BIR), a pathway that requires only one DSB end but that induces long tracks of loss of heterozygosity (Batté et al. 2017). To avoid excessive ssDNA formation that would limit homology search and faithful recombination outcomes, the extent of resection needs to be kept under tight control.

### Resection is regulated at multiple levels

An increasing amount of data indeed shows that resection is regulated at multiple level, ranging from direct modification of the nuclease enzymatic activities or the nuclease protein level to the modulation of their recruitment or efficiency by the chromatin context.

Resection is first regulated by checkpoint activation. At uncapped telomeres in yeast, phosphorylation of Exo1 by the checkpoint kinase Rad53 inhibits its nuclease activity and prevents accumulation of single-stranded DNA (Jia et al. 2004; Morin et al. 2008). In mammals, a negative regulation of Exo1 is also observed at double-strand breaks induced by IR or replication inhibitors. This negative feedback loop is triggered by the ATR-dependent phosphorylation of Exo1 that leads to its degradation and limits hyper-resection and genomic instability (Tomimatsu et al. 2017). The Sae2^CtIP^ protein is also tightly regulated in several species both at the transcriptional and through post-translational modifications (for a review Andres and Williams 2017). Among post-translational modifications, phosphorylation by CDKs is critical to initiate resection in both human and *S. Cerevisiae* (Huertas et al. 2008; Huertas and Jackson 2009). In addition, Sae2^CtIP^ can be acetylated in both budding yeast and human but with different consequences. CtIP acetylation by SIRT6 initiates HR, whereas Sae2 acetylation has been proposed to shunt it into autophagy-mediated degradation (Kaidi et al. 2010; Robert et al. 2011). More recently, the cytosolic form of a mitochondrial metabolic enzyme, the Fumarase, has been shown to be required to maintain Sae2 protein level, whereas this is linked to autophagy remains to be deciphered (Leshets et al. 2018).

A number of recent studies also showed that end resection is finely modulated not only at the level of nucleases and DNA processing enzymes activities, but also through the presence of functional or structural “barriers.” Notably, in both yeast and mammals, the checkpoint protein Rad9^53BP1^ is constitutively bound to chromatin through the interaction between its Tudor domain and H3K79me (van Leeuwen et al. 2002; Huyen et al. 2004; Giannattasio et al. 2005; Wysocki et al. 2005; Grenon et al. 2007). This interaction is further strengthened around DSB sites through interaction of its BRCT domain with DSB-induced γH2A (Javaheri et al. 2006; Hammet et al. 2007). This tight Rad9^53BP1^–Chromatin association has been proposed to act as a barrier to the resection activity of Sgs1–Dna2 possibly by reducing the association of Sgs1 to DSB ends (Lydall and Weinert 1995; Lazzaro et al. 2008; Ferrari et al. 2015; Bonetti et al. 2015). Rad9^53BP1^ is also recruited by Dbp11^TopBP1^, forming a complex that restrains Dna2-mediated nucleolytic processing (Granata et al. 2010; Pfander and Diffley 2011; Villa et al. 2018). This seems conserved in human cells, where TOPBP1 stabilizes 53BP1 to the sites of damage to inhibit DSB resection (Cescutti et al. 2010; Zimmermann et al. 2013; Chapman et al. 2013; Ochs et al. 2016; Liu et al. 2017) and in *S. pombe* where the 53BP1 orthologue, Crb2, specifically inhibits the RecQ-helicase-dependent long-range resection pathway (Leland et al. 2018). In both *S. pombe* and mammalian cells, another resection inhibitor, Rev7 seems to be at play although its mechanism of action is unknown (Boersma et al. 2015; Xu et al. 2015; Leland et al. 2018). On the opposite, Rad9^53BP1^-mediated resection inhibition is counteracted by the Slx4-Rtt107 scaffold that compete for the interaction with Dbp11 and by the Fun30^SMARCAD1^ chromatin remodeler (Chen et al. 2012; Costelloe et al. 2012; Eapen et al. 2012; Dibitetto et al. 2016; reviewed in; Shimada and Gasser 2017).

Therefore, resection is both positively and negatively regulated by chromatin interacting factors to generate enough substrate for efficient search and pairing while avoiding excessive resection and 3’ssDNA loss that would limit homology search.

### Chromatin structure regulates HR

Two main chromatin structures are usually distinguished, a lightly compacted and transcriptionally active euchromatin and a compacted and transcriptionally silent heterochromatin. However, recent genome wide analyses revealed a more complex pattern in most organisms, with non-expressed genes in euchromatin and various repressive chromatin structures defined by different histone modifications and histone binding proteins (Filion et al. 2010; Li and Reinberg 2011; van Steensel 2011; Politz et al. 2013; Becker et al. 2017). The examination of DSB repair taking into account these various chromatin environments has only recently been addressed by either genome wide studies mostly assessing euchromatic DSB sites (Aymard et al. 2014) or approaches specifically targeting particular heterochromatic sites (Goodarzi et al. 2008; Peng and Karpen 2009; Noon et al. 2010; Chiolo et al. 2011; Lemaître and Soutoglou 2014; Ryu et al. 2015; Janssen et al. 2016; Tsouroula et al. 2016; Batté et al. 2017).

These studies all show that the initial chromatin folding at the DSB sites, by itself, largely modulates HR. However, the molecular mechanisms at play still await further characterization. While we will discuss the occurrence and regulation of HR in the compact and transcriptionally silent heterochromatin in the next section, we would like to put emphasize on the fact that even euchromatic loci appear to differ in their capacity to perform HR depending on their chromatin structure. Indeed, HR has been shown to be the prevalent repair mechanism for endonuclease-induced DSB sites in transcriptionally active genes in both *S. cerevisiae* and human cell lines while non-coding or silent sequences exhibit a preference for NHEJ (Chaurasia et al. 2012; Aymard et al. 2014). Somehow counterintuitive when considering these data is the fact that the more compact and silent heterochromatin also mainly relies on HR in different organisms. This is notably the case for heterochromatic repeat-rich regions in G2 mouse cells (Beucher et al. 2009; Tsouroula et al. 2016) and in *Drosophila* pericentromeric heterochromatin (Chiolo et al. 2011).

### Euchromatin and heterochromatin have opposite effect on resection

In human cells, the prevalence of HR in transcribed regions stems from the recruitment of the resection factor CtIP mediated by the binding of the LEDGF protein to the active chromatin marker histone H3 tri-methylated on lysine 36 (H3K36me3) (Daugaard et al. 2012). Functionally, enhanced CtIP recruitment, which is known to be critical to initiate resection, would favour the recruitment of Rad51 and the use of HR at active genes (Aymard et al. 2014). Another active histone mark, the acetylation of histone H4 lysine 16 also seems to promote resection and engagement in HR. Indeed, TSA treatment that increases H4 acetylation concomitantly favours the recruitment of the resection factor BReast CAncer 1 (BRCA1) and diminishes the association of the anti-resection factor 53BP1 to FokI induced DSBs (Tang et al. 2013). Actively transcribed reporters that accumulate H4ac before DSB induction also recruit higher level of BRCA1 and lower 53BP1 (Tang et al. 2013). Both H3K36me3 and H4ac seem thus to actively promote resection and engagement in HR.

While active transcription positively impacts on resection, heterochromatin has recently emerged as a negative regulator of this process in some organisms. Indeed, Sir-mediated heterochromatin at subtelomeric DSB has been shown to limit resection and to increase gene conversion efficiency by preventing loss of genetic information in *S. cerevisiae* (Batté et al. 2017). Resection is also regulated by heterochromatin to some extent in other eukaryotes, although the picture is less clear. Resection regulation seems to vary depending on the cell cycle phase and on the repressive chromatin type. On one end, lamina-associated heterochromatin limits DSB resection (Lemaître et al. 2014) as does pericentromeric heterochromatin in G1 cells (Tsouroula et al. 2016). On the other hand, resection seems to occur at normal rates at centromeres in both G1 and G2 cells (Tsouroula et al. 2016). However, resection occurs and seems even more rapid in Drosophila heterochromatin (Chiolo et al. 2011). It is to note that, in these studies, resection was mostly assessed by the visualization of ssDNA binding proteins’ foci such as phosphorylated RPA or ATRIP that may alternatively reflect persistent binding. Notably, in Drosophila, the fastest appearance of ATRIP^ATR^, a protein recruited to resected DNA coated by RPA, could reflect an enhanced recruitment of ATRIP through direct interaction with HP1a which binding increases upon DSB induction of the DSB (Chiolo et al. 2011). Further studies directly evaluating ssDNA amount will be needed to fully understand the molecular events occurring and their consequences for repair.

### Limiting resection in heterochromatin: what functional consequences?

In *S. cerevisiae*, limiting resection at silent subtelomeres clearly restrains resection-mediated loss of the telomeric proximal sequences and prevents mutagenic BIR repair (Batté et al. 2017). Sir-mediated chromatin structure could also participate to telomere capping and limit resection at the terminal TG repeats protecting telomere from unwanted recombination events (Lue and Yu 2017).

More generally, limiting resection in repeated heterochromatic regions should prevent repair through single strand annealing (SSA), thus limiting the loss of repeated sequences (Stark et al. 2004). This would be particularly relevant at centromeric-repeated sequences in mammalian cells which deletion can lead to centromere inactivation (Stimpson et al. 2010). However, both Drosophila and mammalian centromeric heterochromatin seem permissive to resection. Strikingly, in these cases, heterochromatin negatively regulates the assembly of the Rad51 recombination filament required for strand invasion. In both instances, resected DSBs have been shown to exit the heterochromatin domains prior Rad51 binding (Chiolo et al. 2011; Tsouroula et al. 2016). It is likely that the spatial separation between DNA end resection and homology search prevents illegitimate HR between repeats of different chromosomes that cluster in these domains.

## Conclusions

**Fig. 1:**
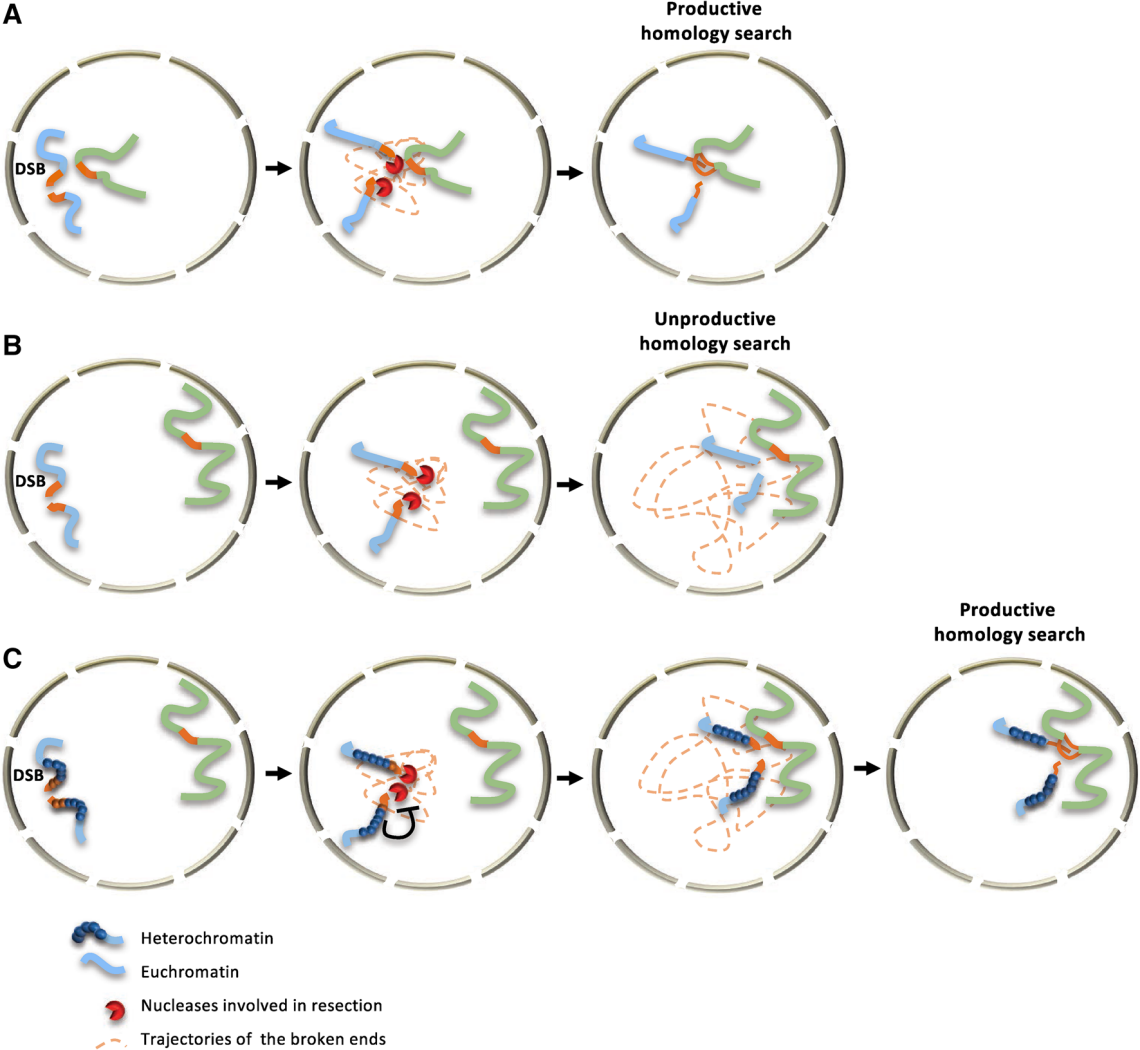
**A** When a DSB occurs on a locus which is in close spatial proximity to the recombination donor, the two sites can encounter after resection has unmasked homologous sequences but prior elimination of the homologous sequence. The homology search process is then productive and recombination repair successful. **B** If the DSB and the recombination donor are too distant, resection will shrink DSB flanking sequences eliminating homologous sequences prior encounter with the donor locus. DSB and global genome-induced mobility can eventually favour timely encounter. **C** The limitation of resection progression by a compact chromatin structure can provide the time for the moving DSB to find the homologous donor and allow a productive homology search

The recent examination of DSB repair taking into account nuclear and chromatin organization, now allows an integrated picture of the steps limiting HR. Although the first limitation has long been known to be the search for a distant homologous matrix to copy (Barzel and Kupiec 2008), it now appears that the distance between the DSB and its repair template is not the sole limiting factor. Indeed, depending on the chromosomal context, resection may act as a countdown that limits the time for the break to explore the entire nucleus (Fig. 1). Soon after DSB occurrence, a race is engaged to cover the distance toward a homologous sequence prior the vanishing of its surrounding sequences. Along the way, unproductive invasions of homologous sequences followed by flap endonuclease processing or the resection machinery itself are ticking the clock. In that instance, both limiting resection and increasing the mobility of the genome may be successful tools to win the homology search race. Chromatin structure, more than being a simple obstacle to DNA repair now appears as a major regulator of HR. Heterochromatin acts at several levels ranging from resection regulation to the control of recombination filament assembly. Although this predicts that not all sequences will be equal in this race, how the mobility varies with chromatin structure and modulate HR efficiency remains to be deciphered.

These new concepts have been and will be key to improve genome-editing strategies. The notion that homology search is limited by the distance between the DSB site and the recombination donor was at the basis of the tethering of the donor DNA to Cas9 and more recently of the targeting of the donor to Cas9 breaks by the Fkh1 protein from *S. cerevisiae*, two approaches that significantly increased genome-editing efficiency (Ma et al. 2017; Gu et al. 2017; Savic et al. 2017; Roy et al. 2018). In some cases, modulating mobility and/or resection efficiency may also be key to successful Cas9 editing (Charpentier et al. 2018).
